# Process Evaluation of an Online SUpport PRogram for Older Hearing Aid Users Delivered in a Cluster Randomized Controlled Trial

**DOI:** 10.3389/fmed.2021.725388

**Published:** 2021-10-22

**Authors:** Janine F. J. Meijerink, Marieke Pronk, Birgit I. Lissenberg-Witte, Vera Jansen, Sophia E. Kramer

**Affiliations:** ^1^Otolaryngology - Head and Neck Surgery, Ear & Hearing, Amsterdam Public Health Research Institute, Amsterdam University Medical Center (UMC), Vrije Universiteit Amsterdam, Amsterdam, Netherlands; ^2^Epidemiology and Data Science, Amsterdam University Medical Center (UMC), Vrije Universiteit Amsterdam, Amsterdam, Netherlands; ^3^Schoonenberg HoorSupport, Rotterdam, Netherlands

**Keywords:** process evaluation, communication program, eHealth, hearing aid users, implementation, hearing aid dispensing practice, self-management

## Abstract

**Objectives:** To evaluate the process of implementing a web-based support program (SUPR) for hearing aid users in the Dutch dispensing setting in order to allow interpretation of the randomized controlled trial's results (positive effects on hearing-aid related outcomes; no effects on psychosocial outcomes).

**Design:** Measures: context of implementation, recruitment, SUPR's: reach, implementation fidelity, dose delivered, dose received, satisfaction, and benefit. Data collection: quantitative and qualitative.

**Study Sample:** One hundred thirty-eight clients (mean age 68.1 years; 60% male) and 44 dispensers completed questionnaires. Five clients and 6 dispensers participated in interviews and focus groups.

**Results:** Clients and dispensers were generally satisfied with SUPR's usefulness. SUPR-videos were watched by 7–37% of the clients. Around half of the dispensers encouraged clients to watch them or informed them about SUPR. Some clients found the SUPR-materials suboptimal, and changes in personnel and limited dispenser-training were barriers acting on a contextual level.

**Conclusions:** This study identified several factors that contributed to the success of SUPR. Others factors, acting on various levels (e.g., intervention material, dispensers, and implementation context), were suboptimal and may explain the absent psychosocial effects. The identified factors are important to consider in further development of SUPR, and in other web-based support programs.

## Introduction

Hearing aid (HA) fitting is a central component of aural rehabilitation (AR) (1). HA use has been found to ameliorate the adverse psychological, social, and emotional effects of hearing loss, thereby improving health-related quality of life (2). Still, HAs cannot restore normal hearing levels (3), and residual activity limitations and participation restrictions often remain (4). This is an important reason why a substantial percentage of HA users (i.e., 3–24%) use them <1 h per day or not at all (5–10). Other factors that contribute to low HA use include difficulties in handling and maintaining the HAs, and feelings of embarrassment and stigma associated with wearing HAs (11).

It has therefore been suggested that AR should use a holistic rather than a biomedical (or impairment) approach. This means that AR should include aspects that go beyond HA fitting, like support in the use of communication strategies, acceptance of hearing loss, and HA handling skills (3, 12). These aspects have been incorporated into several group and individual educational programs developed for adults with hearing loss and are typically offered as an addition to HA fitting (1). They usually provide a combination of training in communication strategy use, instruction in HA management, or counseling aimed at supporting the individual's emotional coping with the consequences of hearing loss (12). While educational group programs are often led by clinicians, individual programs are usually self-directed and supported by audiovisual or written materials (13). In recent years, a promising alternative to deliver these programs has emerged, i.e., *via* eHealth technologies (14). Delivery of communication programs *via* eHealth allows for services that can improve (cost-) effectiveness and access to hearing care (15), because they can be delivered at the intensity the patient prefers, in an automated fashion (with limited efforts for health care professionals), and with a wide reach (16).

An example of an effective web-based educational communication program is the online program by Thoren et al. (17). This program included self-study in hearing anatomy, HAs and communication strategies, professional online interaction, and online contact with peers. The program reduced hearing-related participation restrictions and activity limitations in a sample of experienced Swedish HA users who were recruited *via* local advertisements and the internet (17). More recently, the program was expanded with telephone support and tested in a clinical setting, i.e., in three Swedish hearing clinics (18). That study showed improvements in the use of communication skills at 6 months follow-up (18). Another example of a web-based program is that by Ferguson et al. (19), who created Reusable Learning Objects (RLOs, i.e., short interactive videos) covering information on HA care, communication strategies, and adaptation to wearing HAs. The program was found to be successful in improving HA use and practical HA skills in first-time HA users attending the Nottingham Audiology Service (19). Thus, these findings provide evidence for the effectiveness of web-based interventions for HA users. However, the effects have not yet been evaluated using a real-life research design, and the long-term effects (i.e., up to at least 1-year post-intervention) are unknown.

**Figure 1:**
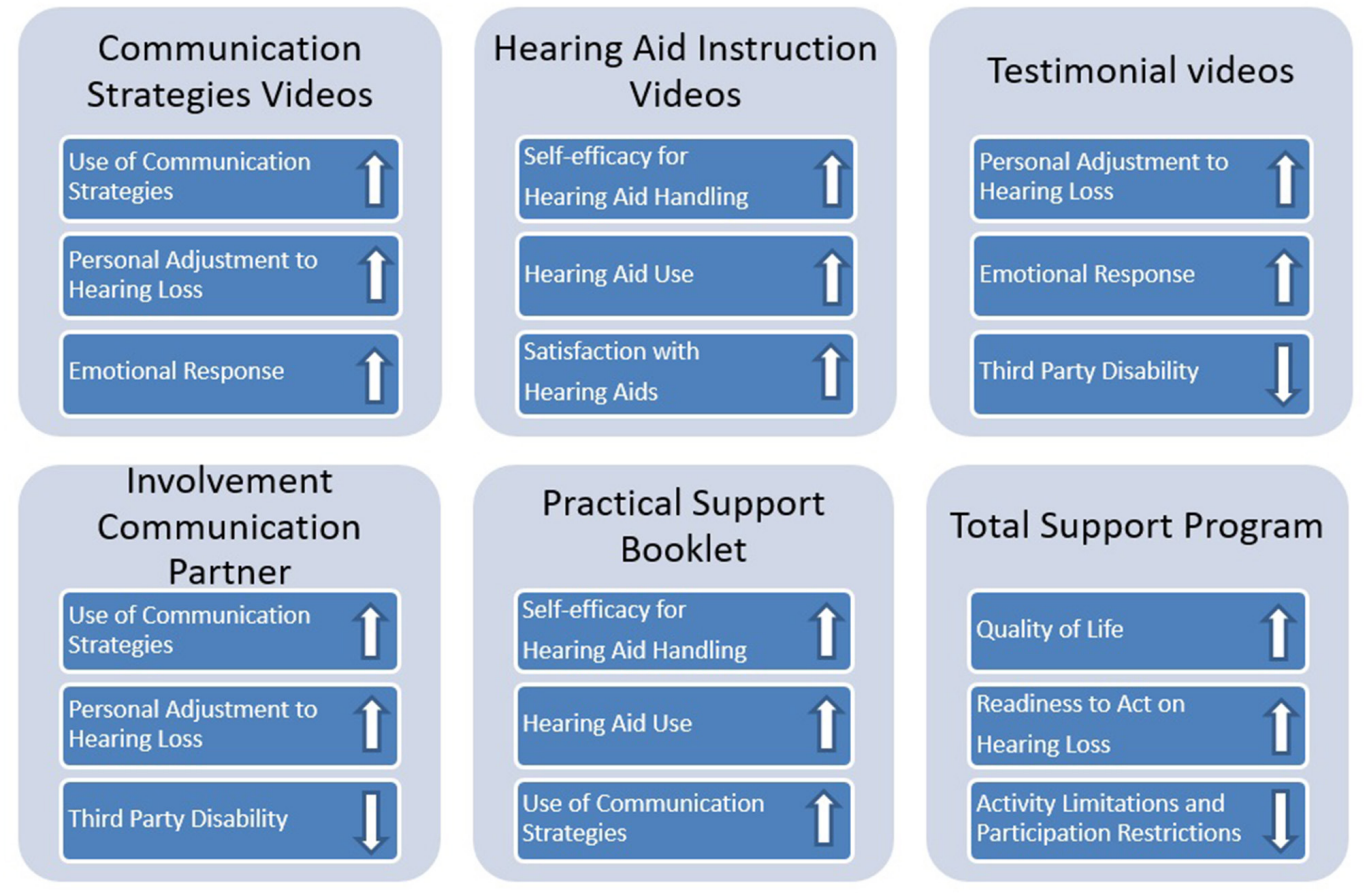
Links between the different SUPR elements and outcome measures. An “up” arrow indicates that the element aimed to improve the particular behavior or appraisal, while a “down” arrow indicates that it aimed to reduce the particular hearing disability.

Our research team recently contributed to the development of a web-based SUpport PRogram (called SUPR) for adult (50+) HA users and their communication partners (CPs) to be offered in a hearing aid dispensing (HAD) setting in addition to a usual HA fitting trajectory. The CP could be any person the client communicates with on a regular basis, i.e., a partner, child, neighbor, or caregiver. SUPR's main aims are to increase HA users' use of communication strategies and a range of secondary outcome measures (see later). SUPR is based on the home education program by Kramer et al. (20), which was shown to be successful in improving quality of life, and communication strategy use at a 6-month follow-up. In 2015, SUPR was created in order to deliver the videos *via* the internet, and the home education program element was supplemented with HA instruction videos and peer testimonials. Figure 1 displays each of SUPR's elements, along with the outcome measures and groups (person with hearing loss or CP) that each of them targeted. The primary outcome included the use of communication strategies by the HA user. All other measures were considered secondary outcomes. All measurement instruments can be reviewed in the effectiveness paper (see below). The full description of the developmental process of SUPR is reported in a lessons learned paper (21).

In a previous study (22), we reported on the effectiveness of SUPR through a cluster randomized controlled trial (cRCT), including 343 clients from 70 HAD practices. The design of the cRCT is described further in the following section (under Materials and Methods) and the study protocol (23). We found no differences in the course of the use of communication strategies between SUPR recipients and controls. For the secondary outcomes, also no effects were found on the psychosocial outcomes (e.g., personal adjustment to hearing loss), but SUPR recipients showed significantly higher self-efficacy for HA handling and HA satisfaction in the long term (i.e., at 1-year follow-up) than controls. Also, SUPR recipients had significantly greater HA use than the controls in the short term (i.e., immediately post-intervention). There were no differences in effects between first-time and experienced clients for any of the outcomes (22). In order to gain more insight into our trial's results and to provide guidance for future use of web-based communication programs like SUPR in clinical practice, we conducted a process evaluation (PE) alongside SUPR's cRCT. Although the PE results were briefly summarized in the lessons learned paper mentioned earlier (21), it did not include the full scope of the results, nor did it include any information on the methodology of the PE study. These aspects are explained in detail in the current paper.

Although an RCT is considered a proper design for establishing the effectiveness of an intervention, they are also criticized for being a “black box,” since it can be difficult to understand why an intervention was a success or failure (24). More specifically, PE studies can help distinguish between implementation and intervention success (or failure), and point to aspects of the intervention that may need to be improved to increase its success. It is therefore generally recommended to perform a PE study alongside an RCT (25, 26). To our knowledge, only two studies that reported on the effectiveness of a web-based AR intervention performed a PE (19, 27). Ferguson et al. (19) evaluated the implementation of RLOs, by assessing accessibility and adherence to, and uptake and acceptability of, the RLOs. A key finding was that all seven RLOs were watched by over 90% of the HA users enrolled (*adherence*) and these were rated as highly useful (*acceptability*). Based on these positive results the authors considered the RLOs a valuable supplement to usual HA care. Recently, Ratanjee-Vanmali et al. (27) conducted a PE to evaluate uptake, patient experience, and satisfaction of a hybrid (web-based and face-to-face) hearing health service model for adult patients with hearing loss. Positive patient experiences and satisfaction were measured, demonstrating the potential of the hybrid service model in clinical practice.

The aim of this PE study was to evaluate the perspectives of SUPR users (clients) and SUPR implementers (HA dispensers; henceforth: dispensers) on eight components, based on Linnan et al. (28) PE framework. These components included: (1) *context* (aspects influencing intervention implementation), (2) *recruitment* (procedures used to attract potential participants), (3) *reach* (target population participating in the intervention), (4) *dose delivered (*components of the intervention delivered to participants), (5) *dose received* (participants' use of and engagement with the intervention materials), (6) *fidelity* (quality of intervention implementation), (7) *satisfaction* with the intervention, and (8) *perceived benefit* of the intervention materials.

## Materials and Methods

### cRCT Description

A full description of the methods and results of the cRCT is reported elsewhere (22, 23). The study was approved by the Dutch Institutional Review Board of the VU Medical University Center Amsterdam. In brief, 34 HAD practices were randomized to the control arm and 36 to the intervention arm. Between February and September 2016, dispensers of these HAD practices recruited clients who were about to enter an HA evaluation period. In total, 343 participants (mean age 68.1 years; SD 8.5; 60% male) were included, and all provided online consent to participate in the study. cRCT outcomes were measured *via* an online survey platform at baseline (T0, when participants had not yet obtained HAs), immediately after SUPR completion (T1, this was 6 months after the client had purchased HAs), 6 months after SUPR completion (T2), and at 12 months after SUPR completion (T3).

### Intervention Description: Care as Usual (Control) and SUPR (Intervention)

#### Care as Usual (Control)

The control group received care as usually provided in the HAD company, i.e., HA fitting only. Care as usual included four appointments with the dispenser. In the first appointment, a screening pure tone audiogram (air conduction only) was performed and the client's goals and wishes related to use of the HAs were discussed. Additionally, clients were advised to appoint a CP and bring them along to the subsequent appointments. The next appointment included full audiometric assessment (i.e., pure tone audiometry including air- and bone conduction and speech audiometry). Based on a protocol as described by Dreschler and de Ronde-Brons (29), the HAs best suited for the client were selected and fitted immediately (if in stock) or at a subsequent appointment. Once the HAs were fitted, the dispenser demonstrated how to insert and clean the HAs and how to change the batteries. This was followed by a HA trial period of ~4 weeks. In this period, the client could decide whether or not to purchase the HAs. If so, a next “purchase appointment” was scheduled. Based on the client's needs, fine-tuning appointments were scheduled during the trial period or after the purchase. Clients were also able to visit the HAD practice every working day during the “service-hour” (4–5 pm), to have problems or questions related to their HAs addressed.

#### SUPR

A full description of the development and implementation of SUPR is described in Meijerink et al. (21). In brief, SUPR is an educational support program consisting of the following elements:

- Practical Support Booklet, which clients received at their first appointment with the dispenser. Clients were asked to take notes related to their specific goals and needs and to describe their experiences with the HA. Additionally, the Booklet contained tips and information on HA use and maintenance, and tips for using communication strategies.
- Seventeen emails in total were delivered over 6 months. The email delivery schedule is presented in Table 1. Eleven emails contained links to educational videos, four contained written communication tips, and two covered information on how to contact the HAD practice customer contact center (see point 3). There were three types of videos: (1) three instruction videos with training modules on the use and maintenance of HAs; (2) five videos with training modules on communication strategies and personal adjustment. These videos demonstrated the difficulties that the main character (a person with hearing loss) experienced in daily life, and how he could successfully counter them by using communication strategies; (3) three testimonials in which peers talked about their experiences with their hearing loss and HAs.
- The option to contact the HAD practice customer contact center *via* email. In email 12 and 16, clients were asked to share their opinion regarding their HAs and their progress with SUPR.
- Involvement of a CP. Similar to the clients in control group, clients were advised to appoint a CP and bring him/her along to all appointments. Clients were additionally instructed to actively involve their CP as much as possible throughout SUPR, for instance by watching the educational videos together. CPs were also encouraged to use the Practical Support Booklet to write down their goals and experiences with the HAs of their loved ones.

**Table 1:** Email delivery schedule of SUPR's online elements.

**Phase**	**Time**	**Email**	**Topic**
Trial period	Week 1	1	Explanation of SUPR (no link)
	Week 2	2	Link to testimonial video: “First experiences with HAs”
	Week 3	3	Link to instruction video: “How to insert HAs”
	Week 4	4	Link to testimonial video: “Together, we hear more”
Purchase of HA	Week 5	5	Link to tips on how to maintain your HAs (text only)
	Week 6	6	Link to instruction video: “How to make life easier and safer – Assistive listening devices”
	Week 7	7	Link to video on communication strategies and personal adjustment: “The Conversation”
	Week 9	8	Link to instruction video: “Maintenance and cleaning of your HAs”
	Week 11	9	Link to video on communication strategies and personal adjustment: “The Birthday Party”
	Week 13	10	Link to communication tips (text only)
	Week 15	11	Link to video on communication strategies and personal adjustment: “On the Streets”
	Week 17	12	Contact with the HA dispenser
	Week 19	13	Link to video on communication strategies and personal adjustment: “At the Doctor's”
	Week 21	14	Link to testimonial video: “Inspired by others”
	Week 23	15	Link to video on communication strategies and personal adjustment: “The Meeting”
	Week 25	16	Link to compilation of tips and contact with the HA dispenser
	Week 27	17	Goodbye and thank you (no link)

*SUPR, support program; HA, hearing aid*.

#### Training of HA Dispensers

To deliver SUPR according to protocol, all dispensers completed an e-learning course and had to attend a 2-day live (in person) training. The aim of the e-learning course was to teach dispensers how to engage the CPs during the clients' appointments in the dispenser practice, and in SUPR. The aim of the first training day was to educate the dispensers about the content of SUPR (how it was developed and what components it covered), and to teach them how to explain the goal and importance of SUPR to their clients and CPs. During the second training day the dispensers practiced how to use the Booklet during a conversation with a client and his/her CP.

### Data Collection and Analysis

We used a mixed-methods research design for this PE applying both quantitative and qualitative methods to collect data on the eight components which are specified in Table 2. Note that data collection only occurred among clients and dispensers who participated in the SUPR arm of the cRCT. The cRCT participants, and thus also the sample for the current study, included both first-time and experienced hearing aid users. However, because we did not find any differences in effects between first-time and experienced clients in the cRCT study (22), these groups were merged for the current study.

**Table 2:** Description of the SUPR-intervention process evaluation parameters, the outcome indicators, and the data sources.

**Parameter**	**Definition**	**PE outcome indicators**	**Data sources**
Recruitment and reach	Recruitment of participants for the study and the proportion of the target population (clients in the intervention group) opting in to receive the SUPR-emails	• Number of invited clients for the SUPR-study • Reasons why clients declined to participate • % of clients consenting • % of clients meeting inclusion criteria • % of clients opting in to receive the SUPR-emails	• Logs of HA dispensers • Researcher records • Database of the HAD company
Context	Factors influencing the implementation of SUPR in the HAD practices	• Dispensers' barriers to and facilitators of SUPR's implementation • % of dispensers who followed the 2-day live-training • The extent to which dispensers: • indicated to have gained enough knowledge to be able to implement SUPR • felt they were sufficiently supported by the HAD headquarters • indicated to be motivated to carry out SUPR in practice	• Focus groups with dispensers • PE questionnaire for dispensers (item 5) • PE questionnaire for dispensers (items 5–7)
Fidelity	The extent to which dispensers executed SUPR as was intended by the developers	• The extent to which dispensers complied with the instruction to: • discuss the clients' goals and experiences written down in the Practical Support Booklet • inform them about SUPR • encourage clients to watch the videos	• PE questionnaire for dispensers (items 2–4)
Dose delivered	The extent to which the different materials of SUPR were delivered	• % of clients to whom the dispensers had handed out the Practical Support Booklet • % of SUPR emails that were delivered to the clients	• PE questionnaire for dispensers (item 1) • Database of the HAD company
Dose received	The extent to which clients actively engaged with, and/or used the intervention materials of SUPR	• *Booklet* • % of clients who received the Practical Support Booklet • The extent to which: clients used the booklet to: • write down their goals and experiences with the HAs • to obtain tips and information • *Videos* • % of clients in the intervention group who started to watch a video • % of clients who watched the full video (of those who started to watch the video) • Average viewing time per video • *CP* • % of clients who reported to have a CP • % of clients who reported to choose a CP to be involved in SUPR and appointments • The extent to which clients indicated their CPs to: • have watched the educational videos • have used the Practical Support Booklet	• questionnaire for clients (item 1) • PE questionnaire for clients (items 2 and 3) • Database of the HAD company and Quadia • Quadia • PE questionnaire for clients (item 6 and 7) • PE questionnaire for clients (items 8, 10, 14, 18)
Satisfaction	Clients' and dispensers' opinions about the different materials of SUPR	• The extent to which dispensers thought SUPR (in general) was useful • Opinions about the different materials of SUPR • The extent to which clients: • thought a particular material of SUPR was useful • would recommend a particular material of SUPR to family, friends, and colleagues • thought that SUPR was worth the trouble	• PE questionnaire for HA dispensers (item 12) • Focus group and interviews with clients and dispensers • PE questionnaire for clients (items 4, 5, 11, 12, 15, 16, 19, 20) • T1 questionnaire of the cRCT (IOI-AI, item satisfaction)
Perceived benefit	Clients' and dispensers' perceived benefit of SUPR	The extent to which clients thought SUPR: • was effective in terms of improving communication, adjustment to hearing impairment, and HA use • helped in a situation where clients most wanted to hear better • The extent to which dispensers thought SUPR was effective in terms of the clients' ability to improve their communication, adjustment to hearing loss, HA use, and the involvement of the CP in the HA trajectory	• PE questionnaire for clients (items 21–23) • T1 questionnaire of the cRCT (IOI-AI, item benefit) • PE questionnaire for dispensers (items 8–11)

*SUPR, support program; HAD, hearing aid dispensing; HA, hearing aid; PE, process evaluation; CP, communication partner; cRCT, cluster randomized controlled trial; IOI-AI, International Outcome Inventory – Alternative Interventions (30)*.

#### Quantitative Data Collection

Four sources for quantitative data collection were used:

1. PE questionnaires for clients (Supplementary Material 1) and dispensers (Supplementary Material 2). The questionnaires were specifically developed for this study. They are based on the PE questionnaires used by Gussenhoven et al. (31). PE data from clients were collected post-intervention. The follow-up questionnaires used in the cRCT were used for this purpose (i.e., the PE questions were added to the outcome measures collected for the cRCT). Recruitment and response rates are described under “Quantitative results, recruitment and reach.” PE data from dispensers were also collected post-intervention. Dispensers of 35 practices allocated to the SUPR arm were invited to fill out the PE questionnaire for dispensers (one HAD was permanently closed at the time of the PE and hence 35 of the 36 practices participating in the cRCT contributed to this PE study). In total, 61 dispensers agreed to participate, of which 44 completed the PE questionnaire. Note that the number of dispensers was higher than 35, as in some practices more than one dispenser was active. A researcher of the study team (J.F.J.M) contacted all HAD practices in the SUPR group to invite the dispensers to fill in the PE questionnaire.
2. Logs of dispensers. Dispensers had to log the number of clients they had invited to participate in the cRCT during the recruitment period. These logs were used to compare them to the number of participants who were eligible and consented to participate. When clients declined to participate, the dispensers also had to log their reasons for decline.
3. Data on clients' online behavior using the HAD company database (a portal storing online client behavior) and the Quadia database (Quadia was the HAD company's supplier of online video content). For each SUPR email, the number of clients clicking the link to the videos' website was available. Note that a click on the link did not mean that these people also actually clicked on the video's “start video” button to watch it. The percentage of clients who started to watch a video relative to participants who clicked on the link in an email was provided by Quadia. By combining the data from the HAD company database and Quadia, we calculated the proportion of SUPR participants who started watching the videos, relative to the total number of participants in the intervention group. To illustrate, if 104 people out of the total of 180 participants in the intervention group clicked on the link in email 3 (HAD company database) and 64% of those 104 clicked on “start video” to watch the video (Quadia), the total proportion of people who started to watch the video linked was 37% [104^*^0,64 = 66.56. (66.56/180)^*^100 = 37%]. Note that in Quadia, start percentages were only available for the total HAD company customer database due to privacy reasons. Hence, the group of clients participating in the cRCT could not be selected as a separate sample. We therefore used the proportion of all clients who had clicked on the “start video” button in the period of the cRCT study (February–June 2017) as a proxy.
4. The “satisfaction” and “perceived benefit” items of the International Outcome Inventory – Alternative Interventions [IOI-AI (30)] administered at t1 in the cRCT study were used to assess satisfaction with, and perceived benefit of SUPR. The item scores ranged from 1 to 5, with higher scores indicating better outcomes.

#### Qualitative Data Collection

In total 96 clients were invited to participate in the qualitative part of the PE study (focus groups or individual interviews). These were clients living in the Dutch provinces Noord-Holland and Groningen. These provinces were chosen to allow variance in participants coming from more urban and rural provinces, respectively. The focus groups took place at a central location in these provinces. Focus groups were preferred, but due to a low response, individual interviews were offered as a secondary option. The invitations were sent by the researchers *via* email. Interested clients were called and informed about the study aims and procedures. In total, 5 clients agreed to participate. Three participated in a focus group, and 2 in individual interviews. Because we aimed to avoid a potential bias of cRCT study participants being influenced by their participation in the qualitative measurements (as data collection was still ongoing at that time), we only invited clients who had recently (<4 months ago) completed SUPR and also met all other in- and exclusion criteria applied in the cRCT (22), but who did not participate in the cRCT. Selecting these clients was possible because at that time, the HAD company had implemented SUPR in all their practices, except the ones participating in the control group of the cRCT.

All dispensers working in the practices allocated to the SUPR-arm were invited to participate in focus groups. Six dispensers agreed to participate and were divided over two focus groups (three participants each). Two focus groups (instead of one) were conducted in order to optimize the range of opinions that would be expressed. Similar to the recruitment of clients, dispensers were firstly approached by email, followed by a telephone call. No in- or exclusion criteria applied.

Semi-structured interview guides were used. Both the individual interviews and focus groups were started by the moderator (J.F.J.M) with the following open-ended question: “What do you think of SUPR?”. This was done to probe issues emerging during the interviews. Subsequently, J.F.J.M. briefly explained and showed the SUPR elements to the participants, to refresh their memories and to facilitate further discussion. Also, neutral, encouraging probes were used (i.e., “Could you tell a bit more about that?”, or “How was this for you?”). The individual interviews and focus groups took 30–60 min. An assistant took notes and observed the group process (focus groups only). The interviews and focus groups were audio-recorded and transcribed verbatim.

#### Quantitative Data Analysis

Questionnaire data were analyzed descriptively by calculating frequencies, percentages, and either means (M) and standard deviations (SDs) (for normally distributed scores), or medians (Meds) and interquartile ranges (IQRs) (for non-normally distributed scores). For all PE-questions with a five-point response scale [“totally disagree” (1) to “totally agree” (5)], the lowest (1) and highest (5), response options were converted into a three-point scale [“disagree” (1) to “agree” (3)]. This was done because the lowest and highest response options were rarely chosen. Data were analyzed using SPSS Statistics version 26.0.

#### Qualitative Data Analysis

Focus group and interview data were analyzed separately for clients and dispensers. Thematic analyses were applied following the six steps as defined by Braun and Clarke (32):

1. Familiarization with data. Transcripts were read multiple times and initial ideas were noted.
2. Generation of initial codes. Commonly occurring patterns across the data set were labeled. This was done by three researchers (J.F.J.M., M.P., and a research assistant) to increase reliability of labeling. Any disagreements were discussed until consensus was reached.
3. Searching for themes. Codes were grouped into potential themes and data (quotes of participants) relevant for that theme were gathered.
4. Reviewing themes. This involved checking the potential themes against the dataset. It included splitting, removing, or combining themes to determine if these properly fitted and explained the data.
5. Defining and naming themes. Step 4 was repeated until clear definitions and names for each theme could be generated.
6. Producing the report. This involved the creation of a story within and across themes to answer the research question.

## Results

### Quantitative Results

#### Recruitment and Reach

Approximately 2,276 clients were invited to participate in the cRCT. This number is an estimation based on the reported number of invited participants by the practices that complied with the protocol to log the number of invitations. Five hundred clients (22%) enrolled themselves for the SUPR study *via* the registration webpage. The main reasons for clients to decline study participation were: not owning a device with an internet connection or an email account, not interested in the study, no time to fill in questionnaires, or perceiving it as too troublesome because of age/illness. Fifty percent (*N* = 248 clients) originated from practices allocated to the SUPR arm. Of these, 180 clients met all inclusion criteria and were included in the cRCT (73%; 180/248). Of these 180 clients, 166 (92%) confirmed their willingness to receive the SUPR emails and 138 of them (77%) completed the PE questionnaire. Baseline characteristics of these 180 cRCT and 138 PE participants are shown in Table 3. There were no statistically significant differences between the two groups for any of the characteristics, indicating that the PE sample was representative for the cRCT intervention group.

**Table 3:** Characteristics of clients who participated in the quantitative part of the PE (column 1) and in the trial (intervention arm only, column 2).

	**PE participants** **(*n* = 138)**	**cRCT participants** **(*n* = 180)**
Male	83 (60)	108 (60)
Age in years, mean (SD)	68.1 (8.0)	68.1 (8.4)
**Marital status**		
Married	101 (73)	130 (73)
Cohabiting	8 (6)	9 (5)
Widowed	16 (12)	24 (14)
Divorced	7 (5)	7 (4)
Single, never married	6 (4)	7 (4)
**Living situation**		
Living together with other people	112 (81)	144 (81)
Living alone	26 (19)	33 (19)
**Educational level**		
Low	29 (21)	38 (22)
Middle	97 (70)	123 (70)
High	12 (9)	16 (9)
**Paid job**		
Yes	25 (18)	39 (22)
No	113 (82)	138 (78)
**Country of birth**		
The Netherlands	127 (92)	162 (92)
Other	11 (8)	15 (9)
Better ear average hearing level in dB HL, mean (SD)	42.8 (10.8)	43.0 (11.7)
**Type of client**		
First-time HAs	90 (65)	116 (64)
Replacement HAs	48 (35)	64 (36)

*Values indicate numbers (%) of participants unless stated otherwise.*

*PE, process evaluation; cRCT, cluster randomized controlled trial; SD, standard deviation; dB HL, decibels hearing level averaged across 1, 2, and 4 kHz; HA, hearing aids*.

#### Context

The PE questionnaire results showed that all dispensers indicated to have followed the 2-day training. Of them, 61% agreed that enough *knowledge* was gained during the training in order to be able to adequately implement SUPR, against 34% who disagreed and 5% who were neutral. Thirty-nine percent agreed that sufficient *support* was provided by the HAD headquarters for them to do so (against 7% who disagreed and 52% who were neutral). Eighty-four percent of the dispensers indicated that they were motivated to carry out SUPR (against 5% who disagreed and 11% who were neutral). Ninety-three percent reported preferring to continue with SUPR in the future (against 2% who disagreed and 5% unwilling to share their opinion).

#### Dose Delivered

In all, 84% of the dispensers reported to have provided the Practical Support Booklet to at least 70% of their clients; 11% reported to have provided the Practical Support Booklet to 50 or 60% of their clients and 5% could not remember to how many clients they had provided the Booklet. The most frequently reported reason for not handing out the Booklet was: “forgot to hand it out.” Regarding the dose delivered of the emails: all 17 emails were delivered to at least 97% of the clients.

#### Dose Received

##### Practical Support Booklet

In all, 78% of the clients reported to have received the Practical Support Booklet, against 8% who had not and 15% who could not remember. Table 4 shows the number of clients who used the Practical Support Booklet to obtain tips and information or to write down goals and experiences with their HAs. The main reason to not (always) write down goals and experiences and to not (always) read the tips and information was “no interest.”

**Table 4:** Use of the practical support booklet among PE participants who indicated having received it (*n* = 107) and the extent to which dispersers (*n* = 44) executed the intervention as was intended.

	**Never**	**Sometimes**	**Always**	**I do not** **know (anymore)**
**PE participants**	***n*** **(%)**	***n*** **(%)**	***n*** **(%)**	***n*** **(%)**
Used the Booklet to obtain tips and information	17 (16)	66 (62)	20 (19)	4 (4)
Used the Booklet to write down goals and experiences	29 (27)	45 (42)	26 (24)	7 (7)
**Dispensers**				
Used the practical support booklet to discuss the clients' goals and experiences	7 (16)	16 (36)	21 (48)	0
Encouraged clients to watch educational videos	8 (18)	16 (36)	19 (43)	1 (2)

##### Educational Videos

**Figure 2:**
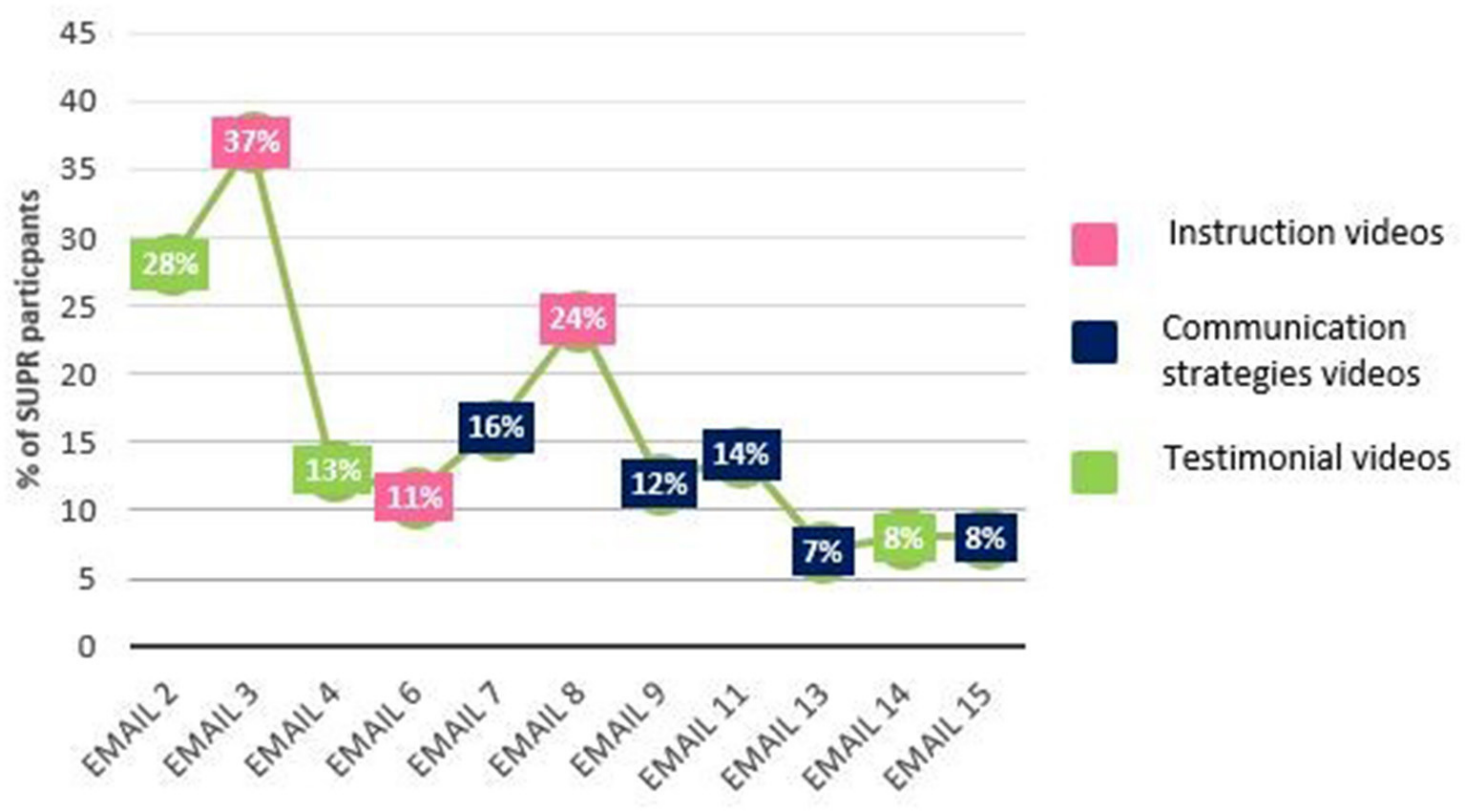
Percentage of cRCT participants in the intervention group (*n* = 180) that started to watch the educational videos (hit the “start video” button) *via* the link provided in the SUPR emails.

Figure 2 presents the percentage of cRCT participants who started to watch the educational videos, i.e., hit the “start video” button *via* the link provided in the SUPR emails. Videos linked to in email 2 (testimonial video “Your first experience with a HA”), 3 (instruction video “How to insert HAs”), and 8 (instruction video “Maintenance and cleaning of HAs”) were watched most often (28, 37, and 24% of the participants started to watch, respectively). The main reason to not have watched all instruction videos (as reported in the PE-questionnaire) was: “I already knew how to handle my HA and/or what kind of assistive listening devices there are.” The main reason to not watch (all) videos on communication strategies and the testimonial videos was “no interest.” Figure 2 shows that overall, with every next email, the proportion of participants watching the video decreased. Table 5 shows the average viewing time per video (column 1), the total video length (column 2), and the percentage of participants who watched the videos until the end (column 3). In general, the percentage of participants watching the entire video was lower for lengthier videos.

**Table 5:** Online behavior of cRCT participants in the intervention group (*n* = 180).

	**Average viewing time**	**Total video length**	**Participants finishing the video^a^**
	**M:S**	**M:S**	**%**
**Instruction videos**			
Email 3: “How to insert HAs”	1:44	1:56	70
Email 6: “How to make life easier and safer – assisting listening devices”	2:19	2:56	60
Email 8: “Maintenance and cleaning of HAs”	3:07	3:46	69
**Videos on communication strategies and personal adjustment**			
Email 7: “Horen en Gehoord Worden” - the Conversation	6:08	9:02	49
Email 9: “Horen en Gehoord Worden” - the Birthday Party	6:57	10:42	42
Email 11: “Horen en Gehoord Worden” - on the Streets	6:18	9:48	40
Email 13: “Horen en Gehoord Worden” - at the Doctor	5:22	7:58	50
Email 15: “Horen en Gehoord Worden” - the Meeting	8:50	18:31	22
**Testimonial videos**			
Email 2: “First experience with HA”	3:19	4:38	62
Email 4: “Together we hear more”	3:01	4:50	48
Email 14: “Inspired by others”	2:49	3:49	61

*M, minutes; S, seconds; HA, hearing aid.*

a *The percentage represents the number of clients who finished watching the video, divided by the number of clients who started to watch it*.

##### Involvement of the CP

In all, 73% of the clients indicated to have a CP and 72% of them had appointed a CP to attend the appointments and be involved in SUPR. Fifteen percent of the clients indicated that their CPs had used the Practical Support Booklet “always” to read tips relevant to them, 35% indicated that their CP had “never” used it, and 35% indicated that their CP “sometimes” used it. Fourteen percent of the clients had forgotten whether or not their CP had used the Booklet. Table 6 shows how clients assessed the involvement of their CP in watching the educational videos. Note that this was only assessed in clients who had indicated to have watched at least one educational video in each category of videos.

**Table 6:** Assessment of CP involvement in watching the educational videos, as viewed by PE participants who had indicated to have a CP (*n* = 100).

	**Did you watch the videos together with your CP?**					
	**Yes, I usually or always watched the videos together with my CP**	**Yes, I sometimes watched the videos together with my CP**	**No, my CP watched the videos at another moment**	**No, my CP did not watch the videos**	**No, and I do not know if my CP watched the videos**	**I do not know (anymore)**
	***n* (%)**	***n* (%)**	***n* (%)**	***n* (%)**	***n* (%)**	***n***
Instruction videos (*n* = 69)	12 (17)	5 (7)	8 (12)	32 (46)	12 (17)	0
Videos on communication strategies and personal adjustment (*n* = 50)	9 (18)	4 (8)	11 (22)	22 (44)	4 (8)	0
Testimonial videos (*n* = 33)	7 (21)	5 (15)	3 (9)	16 (49)	2 (6)	0

*CP, communication partner*.

#### Fidelity

Table 4 shows the number of dispensers who used the Practical Support Booklet to discuss clients' goals and experiences and encouraged clients to watch the online videos. Dispensers' main reason to not (always) use the Booklet was “clients do not fill in their goals and experiences.” The most frequently reported reason for not (always) encouraging clients to watch the videos was “felt no need for it because clients also watch the videos without my encouragement.” In all, 58% of the dispensers declared they had explained their clients the goals of the educational videos. The remaining dispensers had: “not addressed them or only asked for their clients' email address to tell that emails containing links to videos would be sent” (28%), “only asked for the email address” (6%), or “had not told anything about the online part of the intervention” (8%).

#### Satisfaction

Table 7 shows the numbers and percentages of clients who agreed, disagreed, or were neutral, with regard to the usefulness of the different SUPR elements. The mean score on the question asking whether clients would recommend the Practical Support Booklet to others was 4.9 (SD= 3.2) on a rating scale ranging from 0 (not likely) to 10 (extremely likely). For the educational videos the means ranged between 5.7 (instruction videos) and 6.1 (testimonial videos) (SDs 2.7–3.0). The mean score of the IOI-AI “satisfaction” item immediately post-intervention was 3.2 (SD = 1.1) for clients (range 1–5, t1 cRCT follow-up questionnaire). The majority (88%) of the dispensers agreed that the entire SUPR program was useful, against 12% who were “neutral.”

**Table 7:** The extent to which PE participants (*n* = 138) found the SUPR elements useful.

	**Element useful?**		
	**Disagree**	**Neutral**	**Agree**
	***n* (%)**	***n* (%)**	***n* (%)**
Practical support booklet (*n* = 106)	6 (6)	48 (45)	52 (49)
Instruction videos (*n* = 86)	4 (5)	23 (27)	59 (69)
Communication strategies videos (*n* = 67)	2 (3)	25 (37)	40 (60)
Testimonial videos (*n* = 42)	0	16 (38)	26 (62)

#### Perceived Benefit

Table 8 shows the results on the benefit of SUPR perceived from both the clients' and the HA dispensers' perspectives. Clients had a mean score of 3.0 (SD = 1.3) on the IOI-AI item “perceived benefit” immediately post-intervention (range 1–5).

**Table 8:** The extent to which PE participants (*n* = 138) and dispensers (*N* = 44) thought SUPR was effective (on a scale from 1 to 5).

	**According to** **clients**	**According to** **dispensers**
	**Mean (SD)**	**Mean (SD)**
Improvement in communication	3.0 (1.2)	3.5 (0.65)
Improvement in HA use	2.9 (1.3)	3.7 (0.57)
Improvement in personal adjustment to hearing impairment	3.0 (1.3)	3.5 (0.59)
Improvement in involvement of CP in clients' HA trajectory	-	3.5 (0.78)

*SD, standard deviation; HA, hearing aid; CP, communication partner*.

### Qualitative Results

#### Clients

Of the 5 participants, 4 were male. Their mean age was 71 years (SD 2.2). The analyses resulted in the identification of two over-arching themes: (1) Experiences with SUPR elements and (2) Experiences with and views on hearing care. These themes and subthemes are described below.

#### Experiences With SUPR Elements

Participants discussed their experiences with the Practical Support Booklet, the online part of SUPR, the involvement of the CP, and suggestions for improvements. Overall, participants perceived SUPR as a useful addition to the HA fitting process, but their opinions on the usefulness of the specific elements varied.

- Experiences with the practical support booklet

All participants declared that they used the Booklet mainly to obtain information during the HA fitting process and/or used it as a reference afterward. They were generally positive about the content. They found it clarifying and useful, although the content sometimes overlapped with information provided by the dispenser. One participant said: “*If I had questions, then I would read what was going on. And then it would be rather clarifying. That was actually one of the best functions of the Booklet for me.”* One participant reported preferring the Booklet rather than the educational videos because (s)he found the Booklet easier to access. Not all participants used the Booklet to write down their goals and experiences with the HAs. Those who did not, felt it was needless, because they were also able to orally discuss these with their dispenser. Others felt it was like going back to school: “*I think it is a piece of homework. I do have that thing [i.e., the HA] for myself and not for the HA dispenser. And if they want to know how I'm doing, they can ask me, right?*.” One participant explained to write down goals and experiences in the Booklet because then they would come to life more. Another participant said to just obey the request of the dispenser to write down experiences.

- Experiences with the online elements

Most participants indicated that they had watched only some of the educational videos, mainly because they had missed the emails, or felt they were already familiar with the information that would be shown. With regard to the instruction videos, most participants reported that the topic “cleaning of the HAs” was very relevant. Some of the participants who reported to have watched (one of) the videos on communication strategies and personal adjustment found that the situations shown were too exaggerated and too predictable. One participant reported: “*There was one situation that I remember of someone on a birthday party who failed so obviously [in coping with the situation], that you think: yes, I mean, if I'm talking to someone with earplugs in I will also fail. I mean, that was the atmosphere, it was so predictable [that the main character would not cope]*.” Participants who watched (one of) the testimonial vides felt that these did not add anything extra and perceived them as being somewhat condescending. One participant explained: “*This may be a little sensitive, but I had the feeling that the target group was the elderly. The atmosphere was a little bit like, we need to speak to them very distinctly otherwise they will not follow. I found that a bit disturbing.”*

- Suggestions for improvements of the SUPR elements

Some participants suggested making the Booklet more compact by, for instance, removing space to report goals and experiences. One participant recommended dividing the Booklet into two parts: One focusing on hearing loss and everything one can encounter during the HA journey, the second part focusing on maintenance and how to handle HAs. Some participants indicated they would have preferred a more personalized approach with regard to the online part of SUPR, for example by receiving videos that address the person's needs and only on-demand, or by providing access to an online library to allow clients to choose particular videos themselves. One participant proposed to decrease the number of emails and videos: “*The first video you watch with high interest. What is this? Oh this is useful. You watch the second video and then with the third and fourth you think: I think I can take it from here. And then with the fifth video I think: Now I don't want to watch anymore, this is an overkill*.” Another participant advised that the message of the video be transmitted in a shorter time, no longer than 1–2 min. Recommendations for new topics to be included in a future version of SUPR were “getting used to HAs” and “how to deal with background noise.”

- Role of the CP

Most participants reported that their CP did not use the Booklet and/or watch the educational videos. Some participants did not want to involve their CP because they felt that their hearing loss was their own responsibility. To illustrate: “*I never would consider the idea to give her the Booklet. I did show that I received the Booklet, just like you share more things with each other, but not with the idea, this is interesting, you should take a look at it. I mean, her problem was that she noticed my hearing loss, but that has been restored and the other parts, fine, that is your own business*.”

#### Experiences With and Views on Hearing Care

Participants shared their views and experiences on (Dutch) hearing care. They reported on the commercial character of the hearing health care sector, on the dispenser's service level, and the professionalism and quality of the dispenser's supervision.

- Opinion on the hearing sector: commercial character

One participant felt that the mixture of medical and commercial care in the hearing sector was odd and unsatisfactory. (S)he explained that although hearing loss is a medical problem, HA dispensing is a commercial process and (advanced) HAs are expensive (note that in the Netherlands not all types of HAs are reimbursed): “*I can imagine that some people do not have a free choice and are led by their financial possibilities to what hearing care they opt for. I feel privileged that I am not restricted in that sense.” One* participant reported (s)he did not open 90% of the SUPR-emails because (s)he felt that all commercial companies were chasing him/her with emails after (s)he purchased a product: “*I throw away 90% of my email unread because it is all the same… If you buy something you are chased for the rest of your life [with commercial emails]. If I need information, I will look for it myself.”* Another participant did not see the difference between the commercial videos of the HA dispensing practices company and the educational videos of SUPR.

- Opinion on the dispenser's service level

In general, participants were positive about the dispenser's level of service. They especially appreciated the “walk-in” consultation hour between 4 and 5 p.m. allowing them to walk in with any question relating to their HAs. They were also positive about the care offered after the purchase, for instance the provision of the SUPR videos: “*The best thing of the dispenser is the whole trajectory that you will pass through, the diligence, the proper education and also the after sale services, that is also great.”*

- Supervision by the dispenser: professionalism and quality

Most participants were satisfied with the guidance of their dispenser and considered them to be working properly and professionally.

#### Dispensers

Of the six participating dispensers, four were female. Their mean age was 48 years (SD 8.8). On average, they had worked for the HAD company for 16 years. Two main themes related to the implementation of SUPR in clinical practice were identified in their data: (1) Barriers, (2) Facilitators.

#### Barriers to the Implementation of SUPR in Clinical Practice

The barriers dispensers reported on included: Policy changes, training issues, material issues, and clients' non-participation.

- Policy changes

The dispensers reported that policy changes within the company had had a negative impact on staff engagement in providing SUPR. They described several changes in the (national) dispensing system and reorganizations of personnel that had caused resistance and distrust toward providing SUPR in the HAD practice: “*For the past few years there have been enormous changes in this sector…. That has been very hectic and has evoked a lot of resistance… That same year [i.e., 2013] we've had our first real reorganization of personnel, so that caused resistance on resistance.”*

- Lack of training/knowledge

Some dispensers could not remember if they had followed a training on how to implement SUPR, or what the content of the training was. Also, a lack of (recent) training caused the dispensers to forget what the SUPR videos were about. “*… but of course it is not exciting to watch videos of things you are doing [i.e., explaining how to use and clean HAs] every day, so I just looked at the structure so I could see what it was about, but that was a few years ago. Then I didn't think about it anymore and if I'm coming home at night I have other stuff to do, sorry!”*.

- Material issues

Another barrier to discussing the educational videos with clients was the non-physical presence of the material: “*Dispenser 1: So I use it, but my focus is on the Booklet.” Dispenser 2: “Yes, because that is physically available to us… because we see nothing of the online content as soon as they (the clients) sign up for it.”*

- Non-participation of clients

Most dispensers felt that only half of the clients used the Booklet and that this was highly dependent on the motivation of the client. Moreover, they reported that a considerable number of their clients did not use the internet or had difficulties opening the emails and/or videos. Two dispensers reported that this was especially the case in the more rural areas. This had caused the dispensers to “select” the clients they could inform about the online elements: “*I do not explain the internet thing to all of my clients… Sometimes they (clients) will come and say: Yes, I couldn't open it or I couldn't get it sent…. So yeah I'm really looking to the type of client and then I will estimate if they will be able to handle it or not.”*

#### Facilitators of the Implementation of SUPR in Clinical Practice

A range of facilitators to successfully implement SUPR in HAD practices were identified:

- Ease of use of program material

The dispensers believed that the Practical Support Booklet was easy to use, thereby referring to the option to note appointment dates and remarks. They also felt that using the Booklet facilitated the HA fitting process. One dispenser explained: “*If the clients use it [the intervention] well, it is very easy. Because then he will come to the appointments, he fills in the COSI [The Client Oriented Scale of Improvement], he knows what is going on with his hearing loss and what he is missing out on and he also knows what kind of assistive devices exist, so then it can be an enormous support.”*

- Improved quality of care that the dispenser can provide

Dispensers were positive about the possibility to provide additional guidance to their clients. If, for example, the dispensers were running out of time during the appointments they could hand out the Practical Support Booklet: “*If you are really busy, then it is nice that they [the clients] can read it for a while, can let it sink in, and then when they return that you can continue to discuss it further.”*

- Commercial advantage

Some dispensers believed that using SUPR had a commercial advantage to the company (sales-wise). For example, one dispenser explained that several clients had returned to the HAD practice because they had learned about an assistive listening device while following SUPR, and they now wanted to try it out: “*I've had several clients [coming to my HAD practice] who came to purchase an assistive listening device because they had seen it in the videos.”*

- Less returns to HAD practice

A commonly shared feeling among the dispensers was that SUPR led to fewer clients returning with questions about their HAs. They felt that SUPR increased clients ability to handle their HAs (by receiving information about settings, maintenance, and assistive listening devices). One dispenser described*: “A client who does not have to come back to me asking to replace the filter, but can do it by himself, and if that is the result of a video or a Booklet, great, because every little thing that a client can learn from the Booklet and that you don't have to do yourself* … *I think it is really good.”* Fewer clients returning to the HAD practice after the fitting process was perceived as time-saving for both the dispenser and the clients.

The dispensers also believed there were some particular benefits of SUPR to their clients, and this motivated them to work with SUPR. A commonly shared view was that SUPR is highly useful for the clients on several levels, i.e., in the acceptance of hearing loss, and increase in HA use.

- Better acceptance of hearing loss

One dispenser described how the educational videos could increase clients' acceptance of hearing loss: “*And the other one [a testimonial video], I thought it was the one with the couple that talked about their experiences in difficult listening situations. To just hear it live from other HA users, then they [the clients] will watch it with some sort of acceptation.”*

- Increase in HA use

Some believed SUPR would be a trigger and motivation for clients to use their HAs (more often) and that this in turn would lead to higher client satisfaction.

## Discussion

Findings from our cRCT showed that SUPR led to improvements in self-efficacy for advanced HA handling and HA satisfaction in the long term, i.e., 12 months, as well as greater HA use in the short term (directly after completion of SUPR) (22). However, SUPR did not enhance the use of communication strategies (primary outcome). Findings from the current PE study help to explain these outcomes and offer guidance as to how to further develop and implement SUPR, or similar AR interventions, especially those using web-based platforms in clinical practice.

### Interpretation of Main Findings and Transferability to Other Clinical Fields

#### Booklet

The percentage of participants that had received the Booklet from their dispensers seemed reasonably high (78% according to the clients and at least 70% of the clients according to the large majority of the dispensers). However, the proportion of participants that had used the Booklet as intended, especially for the purpose to write down their goals and experiences, may be viewed as suboptimal (42% sometimes did this and 27% never). In general, setting specific goals and formulating expectations explicitly is viewed as a suitable approach for achieving client-centered care and is integral to self-management interventions. This is true for audiology (33–35), but also applies to other chronic conditions (36). For self-management interventions this seems an indispensable element to intervention success (36). With regard to SUPR, it is therefore important that the implementation of using the Booklet is improved, and all dispensers discuss and identify their clients' wishes and needs. Specific training for dispensers may improve this process further. It should be noted that administrating the Client-Oriented Scale of Improvement [COSI (37)] in every client was part of the Dutch HAD protocol at the time of the study. This protocol was enrolled for the whole hearing aid dispensing field in the Netherlands. Unfortunately, we did not collect data on the extent to which this part of the protocol was in fact followed by the clients included in the SUPR study. Thus, we are unsure if goal-setting *via* COSI did occur in parallel to (i.e., separate from) SUPR.

#### Viewing Rates of Online Intervention Elements

Despite the high percentage (92%) of intervention participants who confirmed their willingness to receive the SUPR emails (*reach*), relatively few clients actually engaged with the SUPR elements (*dose received*). The percentage of intervention participants starting the online educational videos ranged from 7 to 37% (emails 13 and 3, respectively), and clearly decreased over time. The videos about communication strategies were placed quite late in the intervention (first one in email 7). Placing key elements of an intervention early on in the program seems an important lesson for self-management programs in general. Regardless, decreasing and low intervention use in general are familiar phenomena for web-based platforms (38, 39): Participants do not take up a new e-intervention, stop using the e-intervention after a certain period, or do not use it according to how it was intended (40). In contrast, Ferguson et al. (19) found that at least 67% of their participants watched all the interactive videos in their multimedia educational program. Although the videos were not all offered *via* the internet (but also *via* DVD for PC and the internet) and participants were specifically requested to watch the videos, this is a much higher percentage than observed in the current study. It may be that using a real-life study design and using web-based elements only with limited motivation from a professional (as in the SUPR-study), resulted in a lower level of engagement.

The quantitative and qualitative data of this PE provided several other possible explanations as to why a relatively low number of participants watched the online educational videos. Many clients who did not watch (all) videos indicated that they had no interest in watching them, or believed they were already familiar with the information provided. This may indicate that not all clients had similar information needs and interests, and suggests that clients may have benefitted more from a personalized approach over the current “one-size-fits-all” approach. Tailoring intervention materials to clients' specific needs is also broadly supported by literature evaluating elements of effective eHealth interventions (41, 42). For instance, as was also expressed by several focus group participants, offering access to an online library to allow selection of specific materials relevant for the person may increase implementation success. This also ties in with the importance of goal-setting: if particular goals were set for a person (e.g., relating to personal adjustment of hearing loss) the program could have been tailored to the person. Another reason for low intervention use might be related to the commercial character and setting in which the intervention was provided, as was suggested in the focus group and individual interviews. Several studies showed that users of an intervention typically believe that information is more trustworthy and reliable when given in an academic rather than in a commercial context (43, 44). This is supported by Preminger et al. (45) who reported that a commercialized approach typically results in low trust in hearing healthcare providers. A third reason for non-adherence may be dissatisfaction with the content of some videos. The quantitative data indicated that around two-thirds of the participants were satisfied with the usefulness of the videos (vs. around one-thirds being neutral, and 3–6% being dissatisfied). The focus group and individual interviews indicated that some participants were dissatisfied with some of the video content as they found the content sometimes too simplistic, which caused irritation. Others felt there were too many videos, or found them too long. An indirect sign that the duration of the videos may have been too long can be deduced from the online behavior data: especially the lengthier videos were not watched fully. In the current study, we did not assess design aspects such as entertainment value and message style (44). We suggest that further research and development should cover these aspects.

The cRCT showed that intervention effects were only found for outcomes related to the HA handling domain and not for outcomes in the psychosocial domain (22). This corresponds with the PE finding that relatively more participants engaged with the HA instruction videos (HA outcomes) than with the testimonial and communication strategies videos (psychosocial outcomes). See also Figures 1, 2. Unfortunately, we were not able to evaluate whether engagement with the intervention elements correlated to the degree of behavior change (i.e., the primary and secondary outcomes of the cRCT), as we could not deduce the start percentages of *individual* study participants from Quadia. Alternatively, the absence of any effects on any of the psychosocial outcomes may indicate that outcomes such as personal adjustment and use of communication strategies require active, interpersonal contact and practice with an actual person (see under Involvement of CP).

#### Implementation Fidelity and Contextual Factors

Finally, we argue that low adherence may have been a consequence of the poor compliance of dispensers with the implementation instructions (i.e., low implementation *fidelity*). Only half of the dispensers complied with the instruction to explain the goals of the videos and recommended their clients to actually watch them (quantitative data). Note that this was despite the relatively high perceived benefit of SUPR for their clients (ranging from 3.7 to 4, on a scale from 1 to 5). The quantitative dispenser data showed that many dispensers expected their clients to watch the videos anyway, or they believed that particular clients would not be the right target group for SUPR (i.e., had no access to a device with an internet connection and/or were able to understand how to use the internet). Also several contextual factors (parameter *context*) such as policy changes, lack of (re-) training, and material issues were perceived by the dispensers as barriers. Across all fields that involve health behavior change, these are all factors typically associated with low implementation success (46–50), as they interfere with the likelihood to perform a new behavior (50, 51). For example, the focus group data indicated this quite explicitly for the forced cuts in personnel that was carried out by the HAD company at the time of the study: the staff felt resistance to the implementation process. Another barrier appeared to be a lack of training (focus group data and quantitative data). Although all dispensers attended the training, they expressed to feel not optimally equipped to carry out SUPR effectively. Sufficient skills, knowledge, and associated with this, motivation may have hampered the successful implementation of SUPR during the study (48, 50). Nevertheless, the large majority of the dispensers expressed that they were motivated to carry out SUPR (84%), and preferred to continue with the program in the future (93%) (quantitative data). These findings are positive and provide a good basis for optimizing the implementation context.

#### Involvement of CP

Twenty-four to thirty-six percent of the clients reported having watched the educational videos together with their CP and 9–22% reported that their CP watched the videos at another moment (quantitative results). As audiology research showed that engaging family members in AR has clear benefits (52, 53), it seems of importance that dispensers encourage their clients to involve their CPs during SUPR. It may be fruitful, for example, to inform them about the specific potential benefits, and explaining how this is supported by scientific research. For example, this could include the following benefits: a family member can assist in the use and operation of HAs (54), encourage an individual to use HAs (55, 56), and decrease hearing handicap by following support programs together, and providing emotional support mutually (57). Because of the low viewing rates by the HA users and the suboptimal CP involvement, we are uncertain if SUPR's communication strategy and testimonial videos could potentially have improved communication strategy use, personal adjustment to hearing loss, and hearing disability. Comparable hearing self-management e-interventions have found positive effects on such outcomes (17, 18), and suggest that interactions or practice with peers or a professional are indispensable and should be integrated in self-management programs. Further research is needed to examine if e-health interventions for chronic conditions like hearing loss can suffice with an individual approach or whether live- interactions with peers or a professional would be needed to increase success. In the latter case, it should be examined whether live involvement of others may be replaced by online, tailored alternatives, such as avatars or discussion fora. In any case, practicing communication and assertiveness skills with others is essential in case of hearing loss, as many skills relate to communication and thus require active and empathetic involvement of the CP. Moreover, involving and practicing with others is important to overcome the social stigmas related to hearing loss, and this may also be important in other chronic conditions.

### Strength and Limitations

A strength of this study is its comprehensiveness. Drawing on a solid research framework (28), eight process indicators were assessed. Self-reported and empirical client data and dispensers' perceptions were analyzed both quantitatively and qualitatively. Such triangulation of methods is known to generally improve quality of data analysis, and we believe that this has also strengthened the current PE. Another strength is the relatively large number of HAD practices (*n* = 36) spread across the Netherlands participating in this study. The practices were purposefully sampled for spread across all provinces, and for spread in the degree of rural/urban areas being represented. Including such a heterogeneous sample increased the likelihood of the results being externally valid for the chain's HAD practices in the Netherlands.

There are also some limitations that must be discussed. First, there was a chance of social desirability bias for dispensers who may have felt the need to provide a favorable image to the researchers or their colleagues of the HAD practices headquarters. We attempted to prevent this bias by guaranteeing the confidentiality of their answers. Moreover, given that certain outcomes were answered in a more negative way than we expected, social desirability does not seem to have played a significant role. Also, clients who responded to the questionnaires might have been positive about SUPR and study participation in general and might have reported above-average engagement. Again as certain outcomes turned out more negative than expected, the results do not point in this direction. Another limitation refers to the inability to quantify how dispensers perceived the usefulness of the specific SUPR materials because we only assessed how they rated the usefulness of the total SUPR intervention. Yet, the qualitative results did provide some insight into their opinions on this topic. A final limitation in the qualitative data collection for both clients and dispensers is that the sample size was small and data saturation could not be accomplished. The limited sample size was due to a low recruitment response. As a consequence, we cannot be certain that the samples represented the general group of clients and dispensers. It is possible that the full scope of factors that had an impact on engagement with the intervention and the implementation outcomes was not fully represented in this study. Nevertheless, the qualitative results provided useful insights into clients' and dispensers' positive experiences with SUPR, as well as the more negative ones.

## Conclusions

We previously reported that adding SUPR to standard clinical HA dispensing care resulted in short-term and long-term improvements in HA outcomes (22). Combined with reasonably high satisfaction and benefit ratings of intervention materials from both clients and dispensers that resulted from the current study, we argue that these results indicate that SUPR can be considered an effective and useful addition to current care provided in HAD practices. Nonetheless, the educational videos were watched by fewer clients than was expected and may explain the absence of any effects found on psychosocial outcomes.

Insights from this PE suggest that tailoring of intervention elements to clients' specific needs, and investing in training facilities to increase clients' and dispensers' use of SUPR's educational videos are important to consider. A recent review on the state-of-the-art eHealth applied in the patient journey underlined the need for systematic analyses of these elements in order to optimize these eHealth services for adults with HAs (14). The lessons learned in this PE will help inform the further development and implementation of an improved version of SUPR, and possibly also help inform other, future eHealth services in the context of HA rehabilitation.

## Data Availability Statement

The datasets presented in this article are not readily available because this has not been applied for in the ethics application (and participants thus did not consent to use their data for that purpose). Requests to access the datasets should be directed to se.kramer@amsterdamumc.nl.

## Ethics Statement

The study was approved by the Dutch Institutional Review Board of the VU University Medical Center.

## Author Contributions

SK and MP conceptualized and designed this PE study. JM collected the data, wrote the first draft of the manuscript, and analyzed the data. MP assisted in analyzing the qualitative data. BL-W provided statistical and methodological advice. All the authors were involved in data interpretation, commented on drafts of the paper, and approved the final manuscript.

## Funding

This project was funded by AudioNova International. The funder had no role in the data analysis and/or the interpretation of data.

## Conflict of Interest

Most of JM's appointment at the Amsterdam UMC as a PhD student on the SUPR project (including carrying out the tasks related to the submitted work), and the design and implementation of the SUPR study were facilitated through a research grant sponsored by Audionova International. MP was employed as a researcher at Schoonenberg HoorSupport (daughter company of Audionova International) for a 6-month period on other research work, received a (co-funding) research grant from Sonova AG (mother company of Audionova International) for other research work, and has been paid for delivering a one-off scientific presentation for Sonova AG. VJ is an employee at Schoonenberg HoorSupport. SK has been paid for delivering a presentation for Sonova AG; no other relationships or activities that could appear to have influenced the submitted work can be reported. The remaining author declares that the research was conducted in the absence of any commercial or financial relationships that could be construed as a potential conflict of interest.

## Publisher's Note

All claims expressed in this article are solely those of the authors and do not necessarily represent those of their affiliated organizations, or those of the publisher, the editors and the reviewers. Any product that may be evaluated in this article, or claim that may be made by its manufacturer, is not guaranteed or endorsed by the publisher.
