# Supplementary material for: Process Evaluation of an Online SUpport PRogram for Older Hearing Aid Users Delivered in a Cluster Randomized Controlled Trial
Source: Front Med (Lausanne). 2021 Oct 22;8:725388. doi: 10.3389/fmed.2021.725388 (PMC8569232; doi:10.3389/fmed.2021.725388)
Supplement: Supplementary file 1 [file Table_1.docx]

**Supplemental Material 1. Process evaluation questionnaire for clients**

[Please note that for the sake of clarity this questionnaire is a simplified version of the original version. This means that all questions on outcomes that were not used for the process evaluation were excluded from this version. Answer options for each question are presented at the end of this questionnaire and indicated with letter symbols (a-i).]

**Practical Support Booklet**

Item 1: Did you receive the Practical Support Booklet?^a^

*If “Yes”  Item 2*

*If “No”, or “I don’t know (anymore)”  Item 6*

Item 2: How often did you use the Practical Support Booklet to write down your goals and experiences with the hearing aids before an appointment?^b*^

Item 3: The Practical Support Booklet also contains tips and information about hearing aids and communication. Examples of these are tips on how to insert your hearing aids, information on how to maintain your hearing aids, and tips about optimal communication. How often did you use the Practical Support Booklet to read about these tips and information?^b*^

Indicate to what extent you agree with the following statement.

Item 4: I think the Practical Support Booklet is useful^d^

Item 5: How likely is it that you would recommend the Practical Support Booklet to others (family, friends or colleagues)?^e^

**Communication partner**

Item 6: In the Practical Support Booklet a communication partner has been described as the person who you talk to or have contact with on a regular basis. Thus, someone with whom you often communicate. Is there someone who fits in this picture and could this be called your communication partner?^c^

*If “Yes”  Item 7*

*If “No”, or “I would rather not tell this”  Item 10*

Item 7: Has this person also been registered as a communication partner in the hearing aid dispensing (HAD) practice?^a^

*If item 1 was “Yes”  item 9*

*If item 1 was “No”  item 10*

Item 8: The Practical Support Booklet also contained information that was relevant to your communication partner. Did your communication partner read or use the Practical Support Booklet?^b^

**Instruction videos**

Item 9: Did you watch the instruction videos (“How to insert your hearing aids”, “This is how your life could be easier and more saver - hearing aid tools” and “Maintenance and cleaning of your hearing aids”)?^f*^

*If “Yes, all three” or “Yes, one or two” and if item 6 was “Yes”  item 10*

*If “Yes, all three” or “Yes, one or two” and if item 6 was “No” or “I would rather not tell this” item 11*

*If “None” or “I don’t know (anymore)” item 13*

Item 10: Did you watch the instruction videos together with your communication partner?^g^

Indicate to what extent you agree with the following statements.

Item 11: I found the instruction videos useful.^d^

Item 12: How likely is it that you would recommend the instruction videos to others (family, friends or colleagues)?^e^

**Videos on communication strategies and personal adjustment**

Item 13: Did you watch the videos on communication strategies and personal adjustment?^h*^

*If “Yes, all five” or “Yes, three or four” or “Yes, one or two” and if item 6 was “Yes”  item 14*

*If “Yes, all three” or “Yes, one or two” and if item 6 was “No” or “I would rather not tell this”  item 15*

*If “None” or “I don’t know (anymore)” item 17*

Item 14: Did you watch the videos on communication strategies and personal adjustment together with your communication partner?^g^

Indicate to what extent you agree with the following statements.

Item 15: I found the videos on communication strategies and personal adjustment useful.^d^

Item 16: How likely is it that you would recommend the videos on communication strategies and personal adjustment to others (family, friends or colleagues)?^e^

**Testimonial videos**

Item 17: Did you watch the videos about the experiences of other hearing aid users?^f*^

*If “Yes, all three” or “Yes, one or two” and if item 6 was “Yes”  item 18*

*If “Yes, all three” or “Yes, one or two” and if item 6 was “No” or “I would rather not tell this”  item 19*

*If “None” or “I don’t know (anymore)” item 21*

Item 18: Did you watch the videos about the experiences of other hearing aid users together with your communication partner?^g^

Indicate to what extent you agree with the following statements.

Item 19: I found the testimonial videos useful.^d^

Item 20: How likely is it that you would recommend the testimonial videos to others (family, friends or colleagues)?^e^

**Effectiveness of SUPR**

Item 21: How effective was SUPR for the improvement of your communication?^i^

Item 22: How effective was SUPR for the improvement of your hearing aid use?^i^

Item 23: How effective was SUPR for the improvement of or adjustment to the potential disabilities you might experience because of your hearing impairment?^i^

**Answer options**

a Yes/No/I don’t know (anymore)

b Never/Sometimes/Always/I don’t know (anymore)

c Yes/No/I would rather not tell this

d Totally agree/Agree/Neutral/Disagree/Totally disagree

e 0 (not likely)/1/2/3/4/5/6/7/8/9/10 (extremely likely)

f Yes, all three/Yes, one or two/None/I don’t know (anymore)

g Yes, usually or always/Yes, sometimes/No, (s)he watched at another moment/No, (s)he did not watch any/No, and I don’t know if (s)he watched any/I don’t know (anymore)

h Yes, all five/Yes, three or four/Yes, one or two/None/I don’t know (anymore)

i 1 (not effective)/2/3/4/5 (very effective)

* If a participant indicated he/she did not or sub optimally used the particular element of SUPR, the following question that was always asked was: “Why not’’? Next participants could choose between predetermined answer categories (which varied per topic), or an open answer category.
