# Supplementary material for: Process Evaluation of an Online SUpport PRogram for Older Hearing Aid Users Delivered in a Cluster Randomized Controlled Trial
Source: Front Med (Lausanne). 2021 Oct 22;8:725388. doi: 10.3389/fmed.2021.725388 (PMC8569232; doi:10.3389/fmed.2021.725388)
Supplement: Supplementary file 2 [file Table_2.docx]

**Supplemental Material 2. Process evaluation questionnaire for hearing aid dispensers**

[Please note that for the sake of clarity this questionnaire is a simplified version of the original version. This means that all questions on outcomes that were not used for the process evaluation were excluded from this version. Answer options for each question are presented at the end of this questionnaire and indicated with letter symbols (a-f).]

**Practical Support Booklet**

Item 1: Think about the period between February 2016 - August 2016. Try to estimate to what percentage of the clients you handed out the Practical Support Booklet to when they started with their hearing aid evaluation period and SUPR.^a*^

The next questions will be about the here and now.

Item 2: How often do you use the Practical Support Booklet to talk about the clients’ goals and their experiences with their hearing aid(s)?^b*^

**Online elements**

Item 3: To what extent do you inform a client about the online part of SUPR?^c*^

Item 4: Do you encourage clients to watch the videos that are offered via the emails?^b*^

**Carrying out SUPR in practice**

All employees participated in a two-day training in content and use of SUPR elements. Indicate to what extent you agree with the following statements.

Item 5: By following this training I gained enough knowledge to properly carry out SUPR in practice.^d^

Item 6: In general I feel sufficiently supported by the hearing aid dispensing headquarters to properly carry out SUPR in in practice.^e^

Item 7: I am motivated to carry out SUPR in practice.^e^

**Effectiveness of SUPR**

Item 8: How effective do you expect that SUPR is in terms of improving a clients’ communication?^f^

Item 9: How effective do you expect that SUPR is in terms of improving a clients’ personal adjustment to the potential disabilities s(he) might experience because of his/her hearing impairment?^f^

Item 10: How effective do you expect that SUPR is in terms of improving a clients’ hearing aid use?^f^

Item 11: How effective do you expect that SUPR is in terms of improving the involvement of a clients’ communication partner during the hearing aid trajectory?^f^

**Satisfaction**

Item 12: I found SUPR useful.^e^

**Answer options**

a 0% (Not any client)/10%/20%/30%/40%/50%/60%/70%/80%/90%/100% (To all clients)

b Never/Sometimes/Always/I don’t know (anymore)

c I do not tell the client anything about the online part of SUPR/I do not tell the client anything about SUPR, but I do ask what their email-address is/I do ask what their email-address is and tell that emails will be sent with links to videos/ I do ask what their email-address is and tell that emails will be sent with links to videos. In addition, I explain the goals of the videos.

d Totally agree/Agree/Neutral/Disagree/Totally disagree/I did not engage in this training

e Totally agree/Agree/Neutral/Disagree/Totally disagree

f 1 (not effective)/2/3/4/5 (very effective)

* If a participant indicated he/she did not carry out SUPR according to the protocol (for example: did not provide the Practical Support Booklet to all their clients) the following question that was always asked was: “why not’’? Next participants could choose between predetermined answer categories (which varied per topic), or an open answer category.
